# Systemic therapy for advanced clear cell renal cell carcinoma after discontinuation of immune-oncology and VEGF targeted therapy combinations

**DOI:** 10.1186/s12894-020-00647-w

**Published:** 2020-07-02

**Authors:** Yasser Ged, Ruby Gupta, Cihan Duzgol, Andrea Knezevic, Natalie Shapnik, Ritesh Kotecha, Martin H. Voss, Darren R. Feldman, Oguz Akin, Sujata Patil, Robert J. Motzer, Brian I. Rini, Chung-Han Lee

**Affiliations:** 1 Department of Medicine, Memorial Sloan Kettering Cancer Center (MSKCC), 300 East 66th Street, New York, NY 10065 USA; 2 Department of Medicine, Cleveland Clinic Taussig Cancer Institute, Cleveland, OH USA; 3 Department of Radiology, MSKCC, New York, NY USA; 4 Department of Epidemiology and Biostatistics, MSKCC, New York, NY USA

**Keywords:** Immune-oncology, IO combinations, RCC, VEGF, Survival

## Abstract

**Background:**

Several phase 3 studies reported positive results for combinations of Immune-Oncology (IO) and Vascular Endothelial Growth Factor (VEGF) targeted therapies in patients with metastatic clear cell Renal Cell Carcinoma (ccRCC). However, there are limited data on outcomes to systemic therapy after IO-VEGF combinations.

**Methods:**

A retrospective analysis was performed on patients with metastatic ccRCC treated at the Memorial Sloan Kettering Cancer Center and Cleveland Clinic who initiated systemic therapy post IO-VEGF including combinations with VEGF receptor (VEGFR) tyrosine kinase inhibitors (IO-TKI) and combinations with the anti-VEGF monoclonal antibody bevacizumab (IO-Bev). The study objectives were to evaluate the objective response rate (ORR), progression-free survival (PFS) and overall survival (OS) on systemic therapy post IO-VEGF. RECIST v1.1 criteria were used to determine radiological responses and progression. Survival estimates were evaluated with the Kaplan-Meier methods and the log-rank test from the start of systemic therapy post IO-VEGF to the event of interest.

**Results:**

A total of fifty-nine patients were treated post discontinuation of IO-VEGF regimens which included IO-Bev (*n* = 35; 59%) and IO-TKI (*n* = 24; 41%). Fifty-eight patients (98%) received IO-VEGF regimens as part of a clinical trial. Subsequent therapies included cabozantinib (*n* = 22; 37%), axitinib (*n* = 18; 31%), pazopanib (*n* = 4; 7%), lenvatinib and everolimus (*n* = 4; 7%), mTOR inhibitor monotherapy (*n* = 3; 5%), axitinib and dalantercept (*n* = 2; 3%), sunitinib (*n* = 1; 2%), sorafenib (*n* = 1; 2%), and treatment with agents on unreported clinical trials (*n* = 4; 7%). Patients treated on unreported clinical trials were excluded from the efficacy analysis. Post IO-VEGF, the ORR was 25% and median PFS was 12.0 months (95% CI, 8.2–24.5). Median OS was 24.5 months (95% CI, 12–NE) and 12 months OS rate was 63.3% (95% CI, 48.6–74.9). We observed no differences post IO-VEGF OS when comparing IO- TKI vs IO-Bev (Log-rank *p* = 0.73).

**Conclusions:**

Post IO-VEGF, most patients received VEGFR-TKIs. In this setting, VEGFR-TKIs demonstrated clinical activity and remain a viable option for salvage therapy after progression on IO-VEGF.

## Background

Renal cell carcinoma (RCC) is among the top ten most common cancers worldwide with an annual incidence of approximately 65,000 new cases in the United States [1]. An important landmark in the management landscape of metastatic RCC was the Food and Drug Administration (FDA) approval in 2015 of nivolumab, an Immune-Oncology (IO) agent targeting the Programmed Death-1(PD-1) axis based on the Checkmate 025 phase 3 clinical trial [2]. Since then, preclinical data supported the rationale for developing IO-based combinations [3, 4] to improve the clinical outcomes of single agent IO. Such combination approaches included the dual IO combination regimen of ipilimumab and nivolumab which received regulatory approval by the FDA in 2018 as a first-line therapy in patients with intermediate and poor risk metastatic clear cell RCC (ccRCC) based on the phase 3 Checkmate 214 clinical trial [5]. Other studied combinations are the combinations of IO with Vascular Endothelial Growth Factor (VEGF) targeted therapies (IO-VEGF) including IO with VEGF receptor (VEGFR) tyrosine kinase inhibitors (IO-TKI) and IO with the anti-VEGF monoclonal antibody bevacizumab (IO-Bev). Promising results of these combinations were reported in large randomized phase 3 clinical trials in comparison to sunitinib. IMmotion 151 was the first phase 3 clinical trial reporting positive results of a combination of IO with VEGF targeted therapy [6]. In this study the combination of atezolizumab with bevacizumab showed superior progression free survival (PFS) but not in overall survival (OS) compared to sunitinib in previously untreated metastatic ccRCC with PD-L1 positive disease. KEYNOTE-426 [7] investigated the combination of pembrolizumab with axitinib in the first-line setting in patients with metastatic ccRCC, this combination demonstrated improved OS and PFS regardless of the International Metastatic Database Consortium (IMDC) risk group and PD-L1 status compared to sunitinib. JAVELIN Renal 101 [8] investigated the combination of avelumab with axitinib in the first-line setting and demonstrated improved PFS compared to sunitinib, with immature OS results to date. The positive results of KEYNOTE-426 and JAVELIN Renal 101 have led to the regulatory approval of both combinations (axitinib/pembrolizumab and axitinib/avelumab) in patients with metastatic ccRCC in the first-line setting [9, 10]. Currently, several other novel IO based combinations are under study in metastatic RCC [11–13].

However, outcomes of patients treated with anti-kidney cancer systemic therapy after discontinuation of IO-VEGF combinations remain poorly understood and mainly based on few small retrospective series [14–17]. Herein, we report the clinical outcomes to subsequent systemic therapy in a multi-institutional analysis of patients with metastatic ccRCC who discontinued IO-VEGF combinations.

## Methods

### Study population

Institutional review board approval was obtained to retrospectively identify patients eligible for the study from two institutions, Memorial Sloan Kettering Cancer Center (MSKCC), New York, NY and Cleveland Clinic Taussig Cancer Institute (CCC), Cleveland, OH. Inclusion criteria were patients with metastatic ccRCC who initiated systemic therapy for kidney cancer after discontinuation of IO-VEGF combinations including either IO-TKI or IO-Bev.

Patients demographics, clinical features, IMDC risk status, treatment details, survival outcomes, and genomics data were collected if available. Radiological responses were assessed with the Response Evaluation Criteria in Solid Tumors (RECIST) version 1.1 [18].

### Clinical and statistical endpoints

Patient outcomes were recorded from the start date of second line systemic therapy for kidney cancer after discontinuation of IO-VEGF combinations. OS was calculated from the date of starting the next line of systemic therapy until death, and patients who were still alive at the data cutoff were censored at the date of last follow-up. Best radiological overall response rate (ORR) was determined by RECIST version 1.1. PFS was calculated from the start date of next line therapy to the date of progression. Patients who did not progress were censored at the date of their last scan. The cutoff date for follow-up and survival status was Dec 1st, 2018. Patients enrolled on unreported clinical trials were excluded from the outcomes analysis.

Survival curves were calculated using the Kaplan-Meier method. Studied covariates of interest included prior type of IO-VEGF combination, subsequent type of systemic therapy, and underlying genomic alterations. OS and PFS were compared between groups using the log-rank test, and ORR was compared between groups using the Fisher’s exact test. *P*-values of less than 0.05 are considered statistically significant. All analyses were performed using SAS version 9.4 (Cary, NC).

## Results

### Baseline characteristics

Fifty-nine patients with metastatic ccRCC who received subsequent systemic therapy for kidney cancer after discontinuation of IO-VEGF combinations were identified, including 44 from MSKCC and 15 from CCC. All patients were diagnosed with kidney cancer between 6/2013 and 10/2018. Median age at diagnosis was 55 years (range: 33–75 years; Table 1). All patients received IO-VEGF combinations in the setting of a clinical trial with the exception of one patient. Forty-two patients (71%) received IO-VEGF combinations in the first-line setting and 17 patients (29%) in the second-line setting. Prior IO-VEGF combinations included IO-TKI (*n* = 24; 41%) and IO-Bev (*n* = 35; 59%). Nearly all patients (*n* = 55; 93%) discontinued IO-VEGF combinations due to disease progression with a median time from stopping IO-VEGF combinations to starting next line of systemic therapy of 28 days (range: 3–574 days).

**Table 1:** Baseline characteristics of patients with metastatic ccRCC treated with subsequent therapy after discontinuation of IO-VEGF

Patient and tumor characteristics	Entire cohort (***N*** = 59)	^**a**^Patients included in theefficacy analysis (***N*** = 55)
Age at diagnosis (years) – median (range)	55 (33–75)	55 (33–75)
Male gender	46 (78%)	43 (78%)
Prior nephrectomy	56 (95%)	52 (95%)
Prior IO-VEGF combination by category		
IO-Bev	35 (59%)	33 (60%)
IO-TKI	24 (41%)	22 (40%)
Prior IO-VEGF combinations by regimen		
Atezolizumab and bevacizumab	34 (58%)	32 (58%)
Avelumab and axitinib	12 (20%)	11 (20%)
Pembrolizumab and lenvatinib	8 (14%)	7 (13%)
Pembrolizumab and pazopanib	2 (3%)	2 (3%)
Pembrolizumab and axitinib	1 (2%)	1 (2%)
Nivolumab and sunitinib	1 (2%)	1 (2%)
Nivolumab and bevacizumab	1 (2%)	1 (2%)
Reason for discontinuation of IO-VEGF		
Progression of disease	55 (93%)	51 (93%)
Toxicity	3 (5%)	3 (5%)
Other	1 (2%)	1 (2%)
Time from discontinuation of IO-VEGF to start of the next line therapy (days) - median (range)	28 (3–574)	30 (3–615)
IMDC risk at the start of next line of therapy		
Favorable	13 (22%)	11 (20%)
Intermediate	35 (59%)	33 (60%)
Poor	11 (19%)	11 (20%)
Post IO-VEGF next line of therapy		
Cabozantinib	22 (37%)	22 (40%)
Axitinib	18 (31%)	18 (33%)
Pazopanib	4 (7%)	4 (7%)
Lenvatinib and everolimus	4 (7%)	4 (7%)
mTOR inhibitor monotherapy	3 (5%)	3 (5%)
Axitinib and dalantercept (Clinical trial)	2 (3%)	2 (4%)
Sunitinib	1 (2%)	1 (2%)
Sorafenib	1 (2%)	1 (2%)
Unreported clinical trials	4 (7%)	–
Number of therapy line post IO-VEGF		
Second	42 (71%)	39 (71%)
Third	17 (29%)	16 (29%)

*Abbreviations*: *IO-VEGF* Immune-Oncology and Vascular Endothelial Growth Factor targeted therapy, *IO-Bev* Immune-Oncology and Bevacizumab, *IO-TKI* Immune-Oncology and Tyrosine Kinase Inhibitor, *IMDC* International Metastatic Database Consortium, *VEGFR-TKI* Vascular Endothelial Growth Factor Receptor-Tyrosine Kinase Inhibitor, *mTOR* Mammalian Target of Rapamycin

^a^Patients enrolled on unreported clinical trials were excluded from the efficacy analysis

At the time of starting subsequent therapy, patients were predominately IMDC intermediate risk with 13 patients (22%) favorable risk, 35 patients (59%) intermediate risk, and 11 patients (19%) poor risk. Most patients were treated with VEGFR-TKI monotherapy (*n* = 46; 78%). VEGFR-TKI based combinations (*n* = 6; 10%) and mammalian target of rapamycin (mTOR) inhibitors (*n* = 3; 5%) were also used. Four patients (7%) treated on unreported clinical trials were excluded from outcomes analysis. The most common VEGFR-TKI monotherapies were cabozantinib (*n* = 22; 37%) and axitinib (*n* = 18; 31%).

### Treatment and survival outcomes

**Fig. 1:**
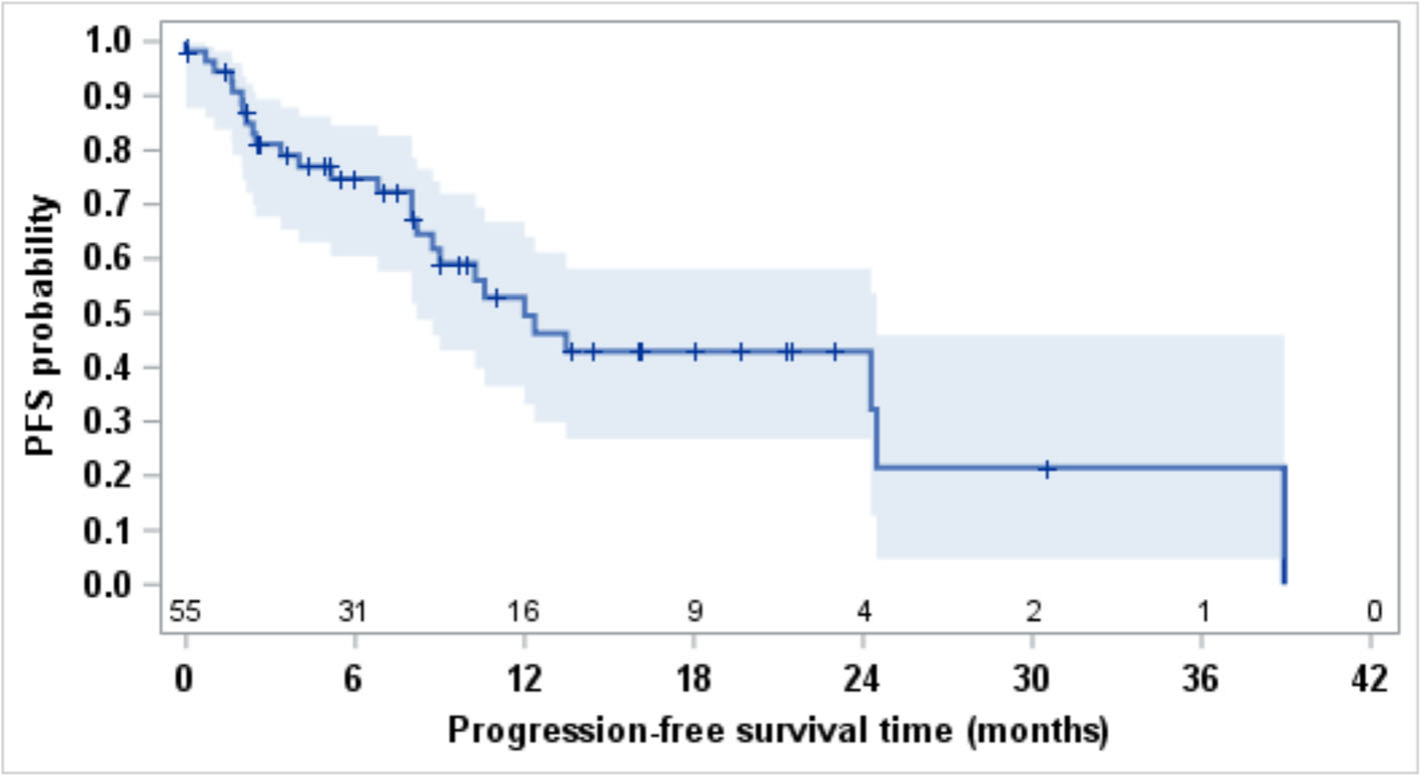
Kaplan Meier curve estimating PFS for 55 patients from the next line of systemic therapy for kidney cancer after discontinuation of IO-VEGF. Median PFS was 12 months (95% CI, 8.2–24.5) and 12-month PFS probability was 49.6% (95% CI, 33.2–64.0). Median follow up time for progression-free survivors was 8.5 months (range: 0, 30). PFS (Progression Free Survival), IO-VEGF (Immune-Oncology and Vascular Endothelial Growth Factor targeted therapy)

**Fig. 2:**
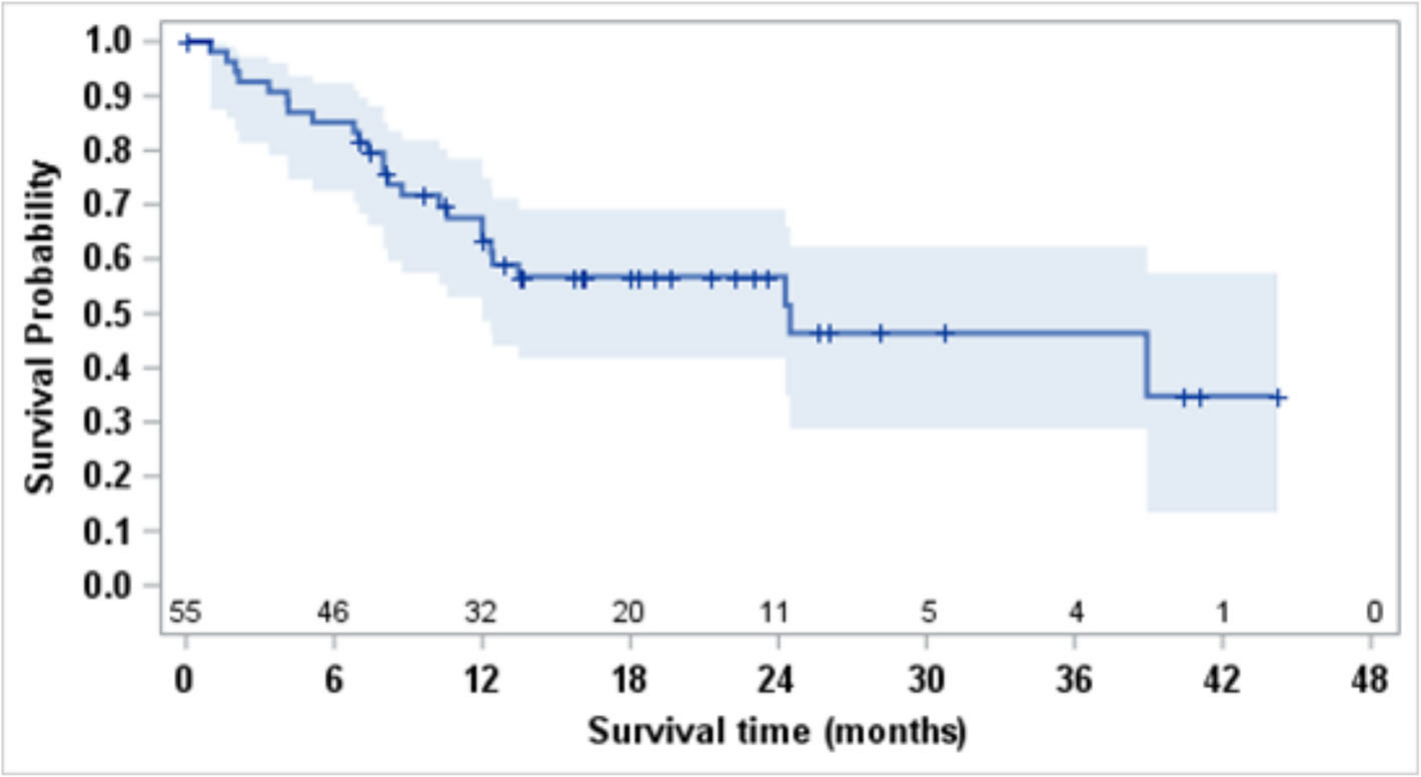
Kaplan Meier curve estimating OS for 55 patients from the next line of systemic therapy for kidney cancer after discontinuation of IO-VEGF. Median OS was 24.5 months (95% CI, 12.0– NE) and 12-month OS probability was 63.3% (95% CI, 48.6–74.9). Median follow up time for survivors was 18 months (range: 0, 44). OS (Overal Survival), IO-VEGF (Immune-Oncology and Vascular Endothelial Growth Factor targeted therapy)

The ORR by RECIST v1.1 on all types of subsequent systemic therapy was 25% (Table 2); all objective responses were partial responses, and the ORR did not differ between IMDC risk groups (*p* = 0.24). Median PFS was 12 months (95% CI, 8.2–24.5; Fig. 1) and varied significantly by IMDC risk stratification (*p* = 0.03; Figure S1). Median follow up time for survival was 18 months (range: 0–44 months) with a total of 25 deaths. Median OS was 24.5 months (95% CI, 12.0–NE; Fig. 2), and the 12-month survival probability was 63.3% (95% CI, 48.6–74.9). Overall survival varied significantly by IMDC risk stratification (*p* = 0.001; Figure S2).

**Table 2:** Objective response rate by RECIST 1.1 assessment for 55 patients

Type of response	N (%)
Complete response	0
Partial response	14 (25%)
Stable disease	28 (51%)
Progressive disease	10 (18%)
Non-evaluable	3 (5%)

*RECIST* Response Evaluation Criteria In Solid Tumors

**Fig. 3:**
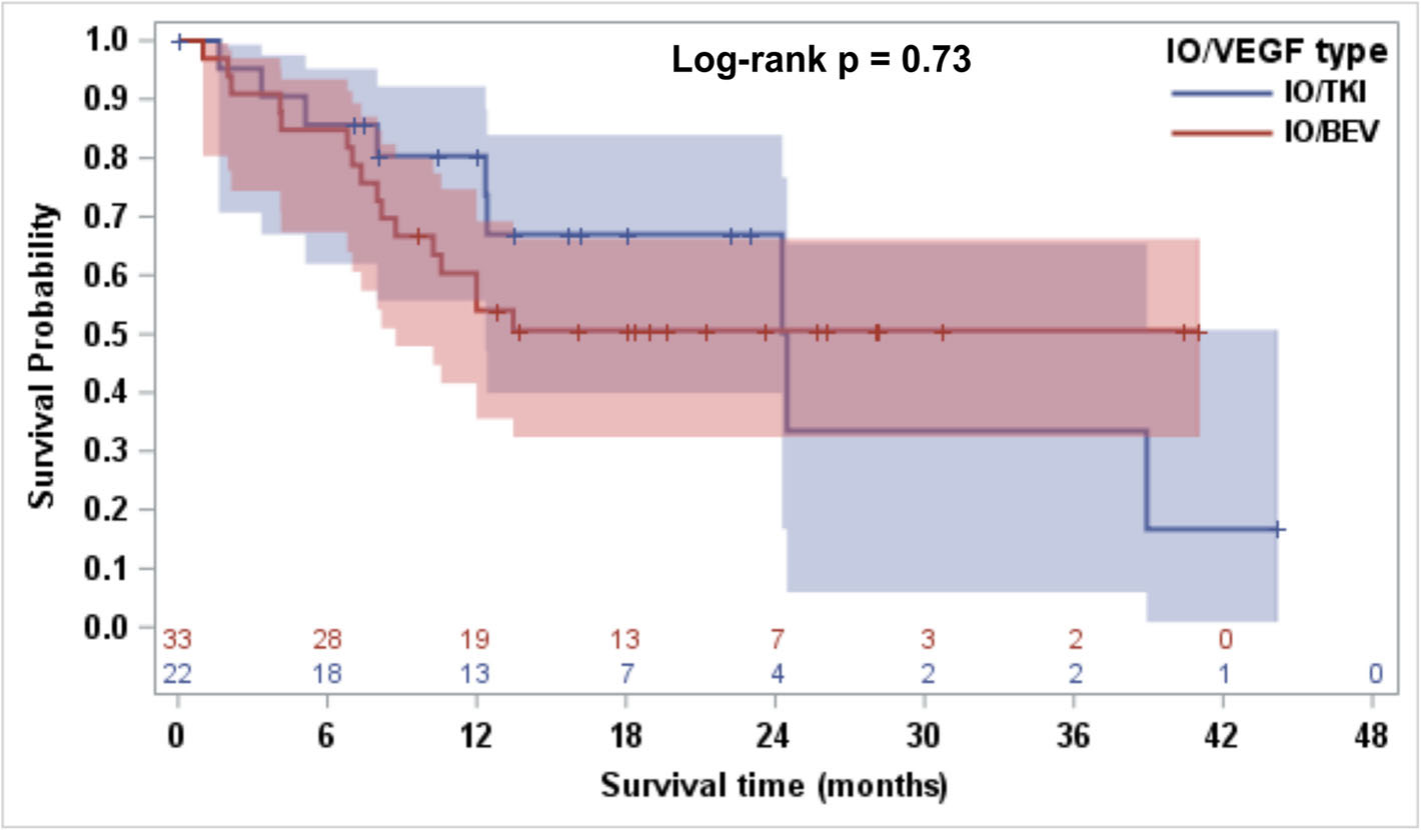
Kaplan Meier curve comparing OS for 55 patients from the next line of systemic therapy for kidney cancer after discontinuation of IO-VEGF by prior received IO-TKI vs. IO-Bev with no statistical difference between the groups (Log-rank *p* = 0.73). OS (Overall Survival), IO-VEGF (Immune-Oncology and Vascular Endothelial Growth Factor targeted therapy), IO-TKI (IO-Tyrosine Kinase Inhibitor), IO-Bev (IO-Bevacizumab)

**Fig. 4:**
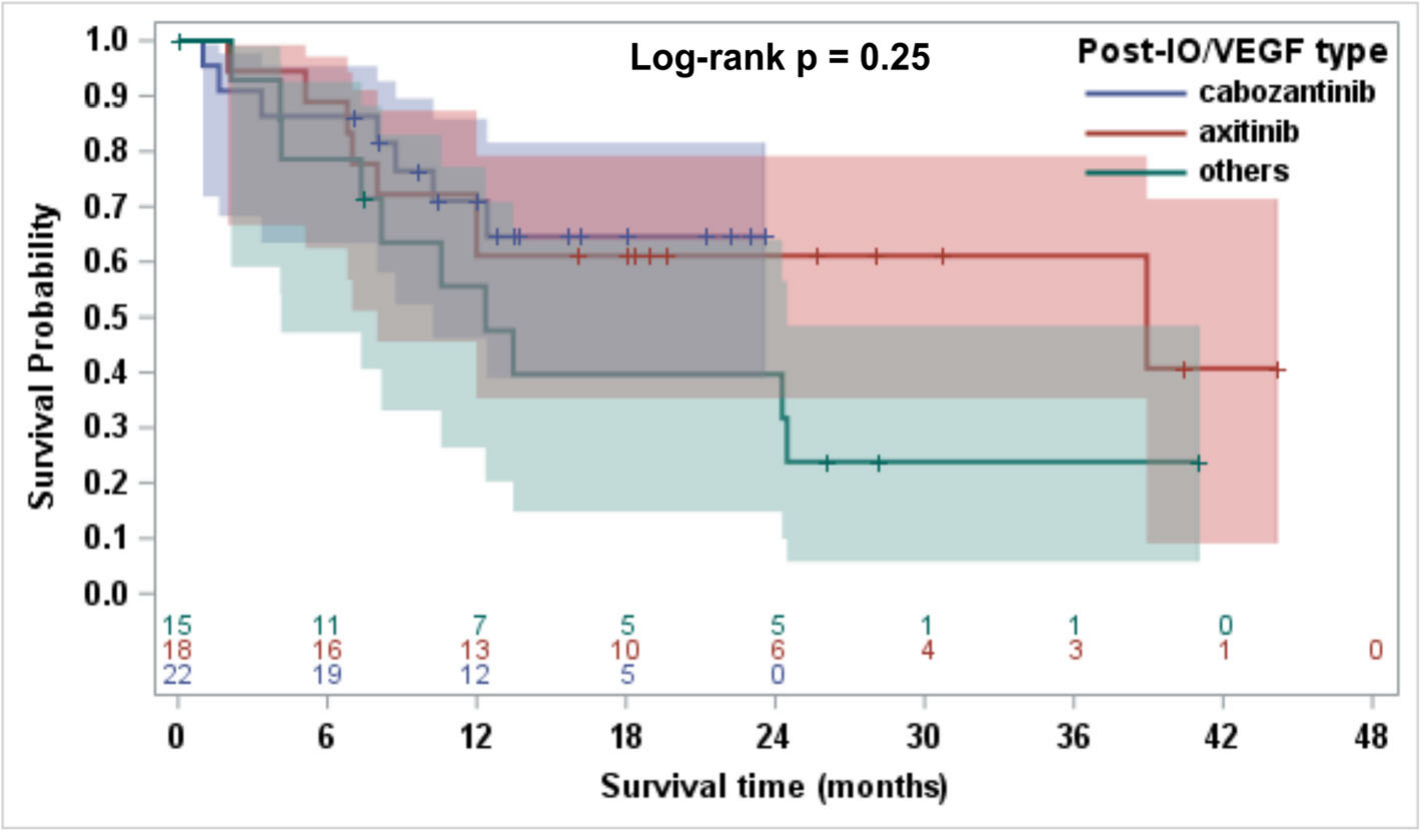
Kaplan Meier curve comparing OS for 55 patients from the next line of systemic therapy for kidney cancer after discontinuation of IO-VEGF by subsequent therapy: Cabozantinib vs. Axitinib vs. Other agents with no statistical difference among the three (Log-rank *p* = 0.25). OS (Overall Survival), IO-VEGF (Immune-Oncology and Vascular Endothelial Growth Factor targeted therapy)

In a subgroup analysis of subsequent therapy comparing patients previously treated with IO-Bev and IO-TKI, no significant differences were seen in ORR (*p* = 0.76; Table S1), PFS (*p* = 0.72; Figure S3) and OS (*p* = 0.73; Fig. 3). Additionally, no significant differences in ORR (*p* = 0.79; Table S2), PFS (*p* = 0.50; Figure S4) and OS (*p* = 0.25; Fig. 4) were observed in the 3-group comparison of subsequent next line therapy (cabozantinib, axitinib and other agents).

## Discussion

The discovery of IO agents [19, 20] has led to remarkable progress in the RCC treatment landscape with the regulatory approval of anti-PD-1 monotherapy (nivolumab) as the first FDA approved IO in RCC [2]. To increase efficacy and overcome resistance mechanisms, preclinical evidence has suggested novel combinatory approaches [3, 4]. The combination of VEGF and PD-1/PD-L1 directed therapies has been studied in several phase 3 clinical trials including the combination of atezolizumab /bevacizumab (IMmotion 151 trial), pembrolizumab/axitinib (KEYNOTE-426), avelumab/axitinib (JAVELIN Renal 101), pembrolizumab/lenvatinib (CLEAR trial), and nivolumab/cabozantinib (CheckMate 9ER trial). Positive results from KEYNOTE-426 [7] and JAVELIN Renal 101 [8] have led to the regulatory approval of pembrolizumab/axitinib [9] and avelumab/axitinib [10] as a standard first-line treatment for patients with metastatic ccRCC, and the results from the CLEAR trial and CheckMate 9ER remain pending. However, despite these promising results, for many patients subsequent therapy remains necessary after discontinuation of IO-VEGF combinations. In this study we conducted a multi-institutional retrospective analysis to examine the efficacy of subsequent systemic therapy after discontinuation of IO-VEGF combinations.

Our study, which was originally presented at the ASCO Annual Meeting, represents the largest study to date of patients who were treated after discontinuation of IO-VEGF combinations [21]. Post discontinuation of IO-VEGF combinations, the majority of patients in our study received cabozantinib or axitinib (*n* = 40; 68%), with the remaining patients receiving other kidney cancer systemic therapies. The activity of cabozantinib and axitinib in previously treated patients with metastatic ccRCC was demonstrated in the METEOR [22] and AXIS [23] randomized phase 3 clinical trials, respectively. The METEOR study [22] examined clinical outcomes of cabozantinib in comparison to everolimus in 658 patients with metastatic ccRCC after progression on prior therapy and reported a median OS of 21.4 months (95% CI, 18.7–NE), PFS of 7.4 months (95% CI, 6.6–9.1) and ORR of 17% for patients treated with cabozantinib. For axitinib, the AXIS study [23] examined clinical outcomes of axitinib in comparison to sorafenib in 723 patients with metastatic ccRCC after progressing on prior therapy and reported a median OS of 20.1 months (95% CI, 16.7–23.4), median PFS of 6.7 months (95% CI, 6.3–8.6) and ORR of 19% for patients treated with axitinib. However, none of the patients on the axitinib arm on the AXIS study [23] received prior IO and only 5% of patients on the cabozantinib arm on the METEOR study [22] received prior IO. In this retrospective study of patients who were previously treated with IO-VEGF combinations, we demonstrated clinical efficacy that compared favorably to historic controls with a median OS of 24.5 months (95% CI, 12.0–NE), median PFS of 12 months (95% CI, 8.2–24.5) and ORR of 25%. These results may be partially explained by the limited number of patients that were available for evaluation. Also of note, 60% of the patients in this cohort were treated with bevacizumab containing combinations, and bevacizumab is less commonly used in the first-line setting; however, outcomes to subsequent therapy were similar when comparing patients with IO-TKI and IO-Bev. It is also possible that patients continued to derive some benefit from prior IO-VEGF combinations even after the discontinuation of the IO agent in view of the short time interval between discontinuation of IO-VEGF and starting the next line of therapy (Median 28 days, range 3–574).

With regards to the activity of VEGF targeted therapy after prior treatment with IO, Ornstein et al. [24] recently reported results from a phase 2 multicentre study on clinical outcomes of 40 patients who were treated with axitinib in an individualized dosing algorithm after receiving IO therapy as the most recent treatment including IO monotherapy or in combination with VEGF targeted therapy with PFS as the primary endpoint. In this study; 71% of patients received IO monotherapy and 5% received IO-VEGF combinations. Axitinib treatment was associated with high clinical activity (45% ORR with 67% of responses lasting > 12 months and a PFS of 8.8 months). Lee et al. recently reported interim analysis of a phase 2 multicentre study which examined the question of salvage therapy with IO based combination after prior IO failure [25] (NCT02501096). In this study, 33 patients who progressed on IO treatment received pembrolizumab and lenvatinib combination, which was associated with promising results in this setting with ORR of 64% and 11.3 months PFS indicating that salvage therapy after IO failure with a combination of IO and VEGF targeted therapy is a feasible approach. Other retrospective studies evaluating the efficacy of VEGFR-TKI agents after failure of front line IO based combination therapies have also reported clinical activity that is possibly impacted by prior therapy. Barata et al. [14] reported a median PFS of 6.4 months (95% CI, 4.4–8.4) and ORR of 29% in a cohort of 33 patients who were treated with VEGFR-TKI after progressing on IO-based combinations (atezolizumab/bevacizumab, ipilimumab/nivolumab, avelumab/axitinib). Auvray et al. [26] reported a median PFS of 8.0 months (95% CI, 5.0–13.0), 12- months survival rate of 54%, and ORR of 36% in a cohort of 33 patients treated with VEGFR TKI after failure of ipilimumab with nivolumab. However, a larger percentage of patients in the aforementioned studies received first generation VEGF-TKIs with sunitinib or pazopanib as the next subsequent therapy (13/33 patients [39%]; Barata et al. and 23/33 patients [70%]; Auvray et al.) compared to our study (6/55 patients [11%]) which may partially explain the differences in outcomes. Furthermore, another retrospective study examined outcomes of 70 patients treated with VEGF-TKI after prior IO therapy (including 36% patients who received IO-VEGF combinations) and showed similar results to our findings with a median PFS of 13.2 months (95% CI, 10.1–NA), 12-months survival rate of 79.6% (95% CI, 70.2–90.3), and ORR of 41% [15].

Some of the limitations of our study include the retrospective design, small sample size, and the relatively short follow-up time. Moreover, the majority of patients included in the efficacy analysis (*n* = 32, 58%) received atezolizumab and bevacizumab combination, which was not associated with OS benefit in the overall population in the IMmotion 151 phase III clinical trial (HR 0.93, 95% CI 0.76–1.14, *p* = 0.47). This combination is currently thought not to be as active compared to IO-VEGF TKI based combinations; thus, this possibly could be driving the favorable outcomes in our study. Furthermore, in view of the multicentre retrospective nature of the study, specific study population data were not collected, including the number of patients with tumors harboring sarcomatoid features, details of metastatic sites, treatment-related toxicity data and the doses of VEGF-R TKI used post IO-VEGF. It is also reasonable to postulate that the favorable outcomes observed in our study might be influenced by the type of therapy received (68% received cabozatinib or axitinib) and that almost all patients were treated with IO-VEGF combinations as part of a clinical trial; suggestive of a favorable population which may limit the applicability of our study findings. Nonetheless, despite these limitations, our study represents one of the largest reports in this setting, providing further evidence on systemic therapy outcomes post-IO-VEGF.

## Conclusions

Our findings indicate continued efficacy of systemic therapy after discontinuation of IO-VEGF combinations which is suggestive of possible continued benefit from prior IO therapy and/or maintaining sensitivity to VEGF directed therapy. Further studies are necessary to validate these findings.

## Supplementary information

**Additional file 1: Figure S1.** Kaplan Meier curve estimating PFS for 55 patients from the next line of systemic therapy for kidney cancer after discontinuation of IO-VEGF by IMDC risk scores (1 = Favorable, 2 = Intermediate, 3 = Poor). PFS varied significantly by IMDC scores (*p* = 0.03). PFS (Progression Free Survival), IO-VEGF (Immune-Oncology and Vascular Endothelial Growth Factor targeted therapy), IMDC (International Metastatic Database Consortium). **Figure S2.** Kaplan Meier curve estimating OS for 55 patients from the next line of systemic therapy for kidney cancer after discontinuation of IO-VEGF by IMDC risk scores (1 = Favorable, 2 = Intermediate, 3 = Poor). OS varied significantly by IMDC scores (*p* = 0.001). OS (Overall Survival), IO-VEGF (Immune-Oncology and Vascular Endothelial Growth Factor targeted therapy), IMDC (International Metastatic Database Consortium). **Figure S3.** Kaplan Meier curve estimating PFS for 55 patients from the next line of systemic therapy for kidney cancer after discontinuation of IO-VEGF by prior received IO-TKI vs. IO-Bev with no statistical difference between both groups (Log-rank *p* = 0.72). PFS (Progression Free Survival), IO-VEGF (Immune-Oncology and Vascular Endothelial Growth Factor targeted therapy), IO-TKI (IO-Tyrosine Kinase Inhibitor), IO-Bev (IO-Bevacizumab). **Figure S4.** Kaplan Meier curve estimating PFS for 55 patients from the next line of systemic therapy for kidney cancer after discontinuation of IO-VEGF by subsequet therapy: Cabozatinib vs. Axitinib vs. Other agents with no statistical difference among the three groups (Log-rank *p* = 0.50). PFS (Progression Free Survival), IO-VEGF (Immune-Oncology and Vascular Endothelial Growth Factor targeted therapy). **Table S1.** Best Objective response rate on next line of therapy post IO-VEGF by prior IO-VEGF combination. **Table S2.** Best Objective response rate on next line of therapy post IO-VEGF by type of next line received.

## Data Availability

The datasets used and/or analysed during the current study are available from the corresponding author on reasonable request.

## Abbreviations

CCC: Cleveland Clinic Taussig Cancer Institute
ccRCC: Clear cell renal cell carcinoma
FDA: Food and Drug Administration
IMDC: International Metastatic Database Consortium
IO: Immune-Oncology
IO-Bev: Immune Oncology-bevacizumab
IO-TKI: Immune Oncology-tyrosine kinase inhibitors
MSKCC: Memorial Sloan Kettering Cancer Center
MSK-IMPACT: MSK–Integrated Mutation Profiling of Actionable Cancer Targets
NGS: Next generation sequencing
ORR: Overall response rate
OS: Overall survival
PD-1: Programmed Death-1
PFS: Progression free survival
RCC: Renal cell carcinoma
RECIST: Response Evaluation Criteria in Solid Tumors
VEGF: Vascular Endothelial Growth Factor
