# Supplementary material for: Systemic therapy for advanced clear cell renal cell carcinoma after discontinuation of immune-oncology and VEGF targeted therapy combinations
Source: BMC Urol. 2020 Jul 2;20:84. doi: 10.1186/s12894-020-00647-w (PMC7331268; doi:10.1186/s12894-020-00647-w)
Supplement: Supplementary file 1 — Additional file 1: Figure S1. Kaplan Meier curve estimating PFS for 55 patients from the next line of systemic therapy for kidney cancer after discontinuation of IO-VEGF by IMDC risk scores (1 = Favorable, 2 = Intermediate, 3 = Poor). PFS varied significantly by IMDC scores (p = 0.03). PFS (Progression Free Survival), IO-VEGF (Immune-Oncology and Vascular Endothelial Growth Factor targeted therapy), IMDC (International Metastatic Database Consortium). Figure S2. Kaplan Meier curve estimating OS for 55 patients from the next line of systemic therapy for kidney cancer after discontinuation of IO-VEGF by IMDC risk scores (1 = Favorable, 2 = Intermediate, 3 = Poor). OS varied significantly by IMDC scores (p = 0.001). OS (Overall Survival), IO-VEGF (Immune-Oncology and Vascular Endothelial Growth Factor targeted therapy), IMDC (International Metastatic Database Consortium). Figure S3. Kaplan Meier curve estimating PFS for 55 patients from the next line of systemic therapy for kidney cancer after discontinuation of IO-VEGF by prior received IO-TKI vs. IO-Bev with no statistical difference between both groups (Log-rank p = 0.72). PFS (Progression Free Survival), IO-VEGF (Immune-Oncology and Vascular Endothelial Growth Factor targeted therapy), IO-TKI (IO-Tyrosine Kinase Inhibitor), IO-Bev (IO-Bevacizumab). Figure S4. Kaplan Meier curve estimating PFS for 55 patients from the next line of systemic therapy for kidney cancer after discontinuation of IO-VEGF by subsequet therapy: Cabozatinib vs. Axitinib vs. Other agents with no statistical difference among the three groups (Log-rank p = 0.50). PFS (Progression Free Survival), IO-VEGF (Immune-Oncology and Vascular Endothelial Growth Factor targeted therapy). Table S1. Best Objective response rate on next line of therapy post IO-VEGF by prior IO-VEGF combination. Table S2. Best Objective response rate on next line of therapy post IO-VEGF by type of next line received. [file 12894_2020_647_MOESM1_ESM.docx]

**Supplementary Material**

**
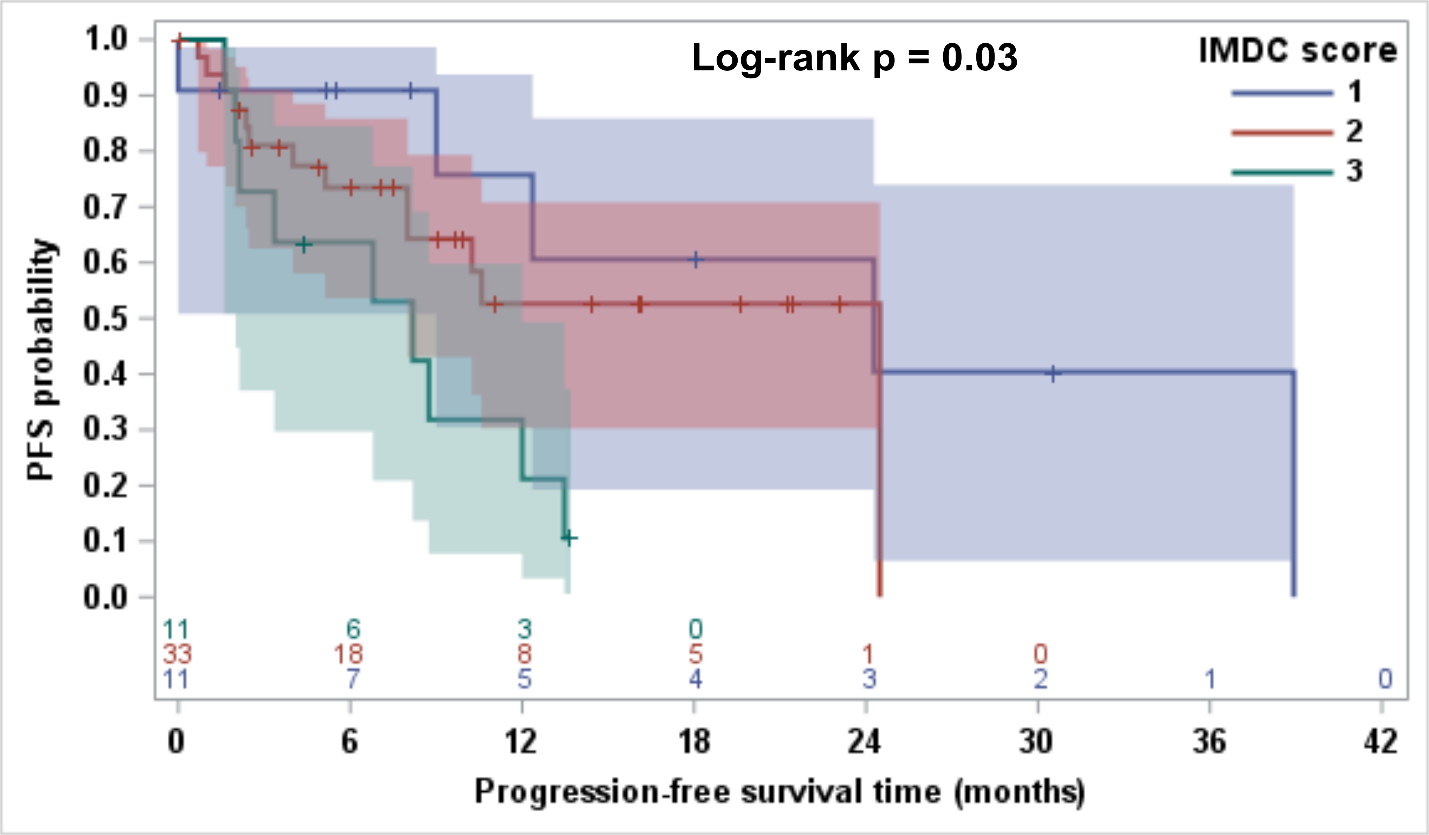
**

**Figure S1**. Kaplan Meier curve estimating PFS for 55 patients from the next line of systemic therapy for kidney cancer after discontinuation of IO-VEGF by IMDC risk scores (1 = Favorable, 2 = Intermediate, 3 = Poor). PFS varied significantly by IMDC scores (p = 0.03). PFS (Progression Free Survival), IO-VEGF (Immune-Oncology and Vascular Endothelial Growth Factor targeted therapy), IMDC (International Metastatic Database Consortium).

**
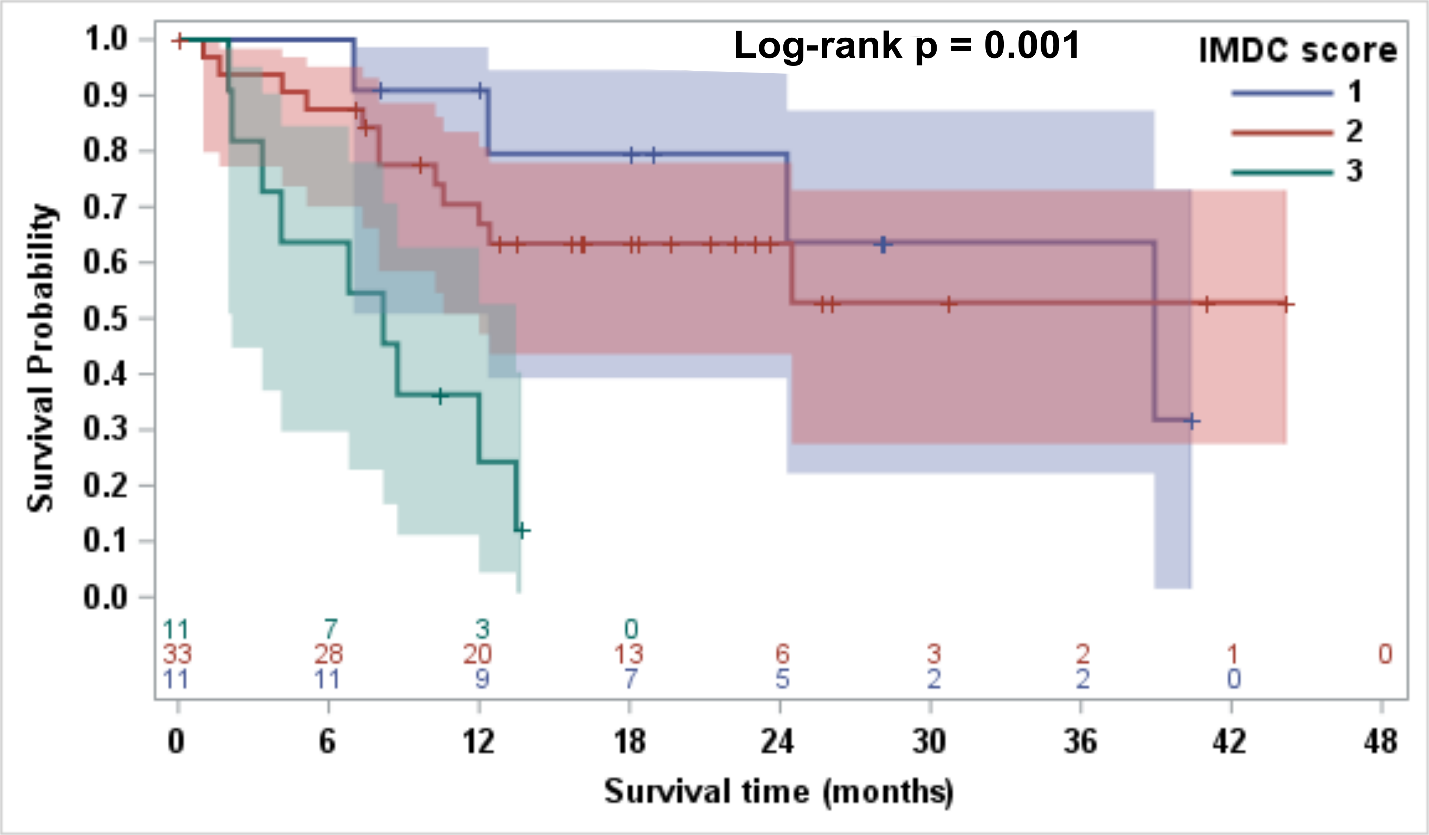
**

**Figure S2**. Kaplan Meier curve estimating OS for 55 patients from the next line of systemic therapy for kidney cancer after discontinuation of IO-VEGF by IMDC risk scores (1 = Favorable, 2 = Intermediate, 3 = Poor). OS varied significantly by IMDC scores (p = 0.001). OS (Overall Survival), IO-VEGF (Immune-Oncology and Vascular Endothelial Growth Factor targeted therapy), IMDC (International Metastatic Database Consortium).


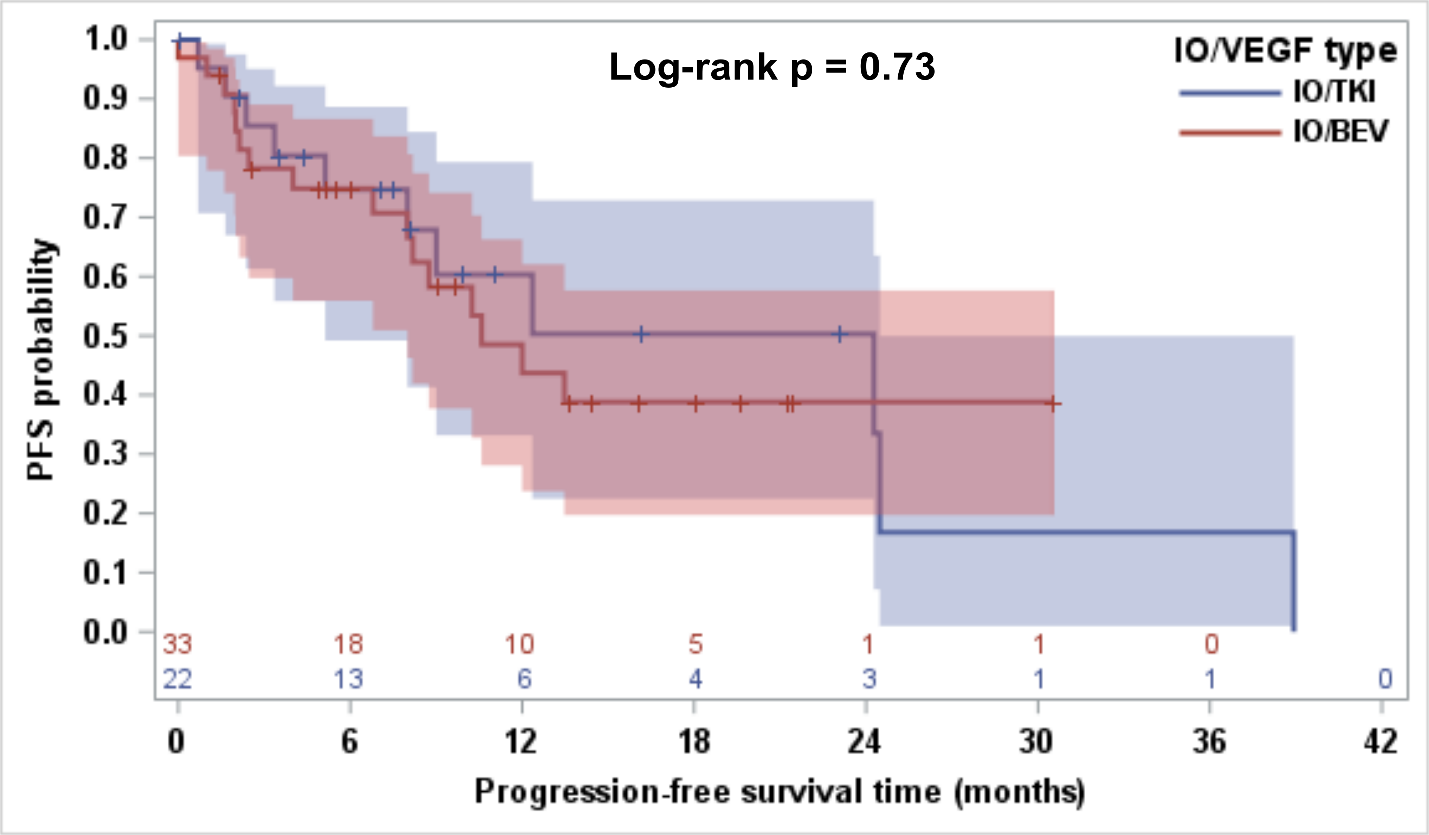


**Figure S3**. Kaplan Meier curve estimating PFS for 55 patients from the next line of systemic therapy for kidney cancer after discontinuation of IO-VEGF by prior received IO-TKI vs. IO-Bev with no statistical difference between both groups (Log-rank p = 0.72). PFS (Progression Free Survival), IO-VEGF (Immune-Oncology and Vascular Endothelial Growth Factor targeted therapy), IO-TKI (IO-Tyrosine Kinase Inhibitor), IO-Bev (IO-Bevacizumab)


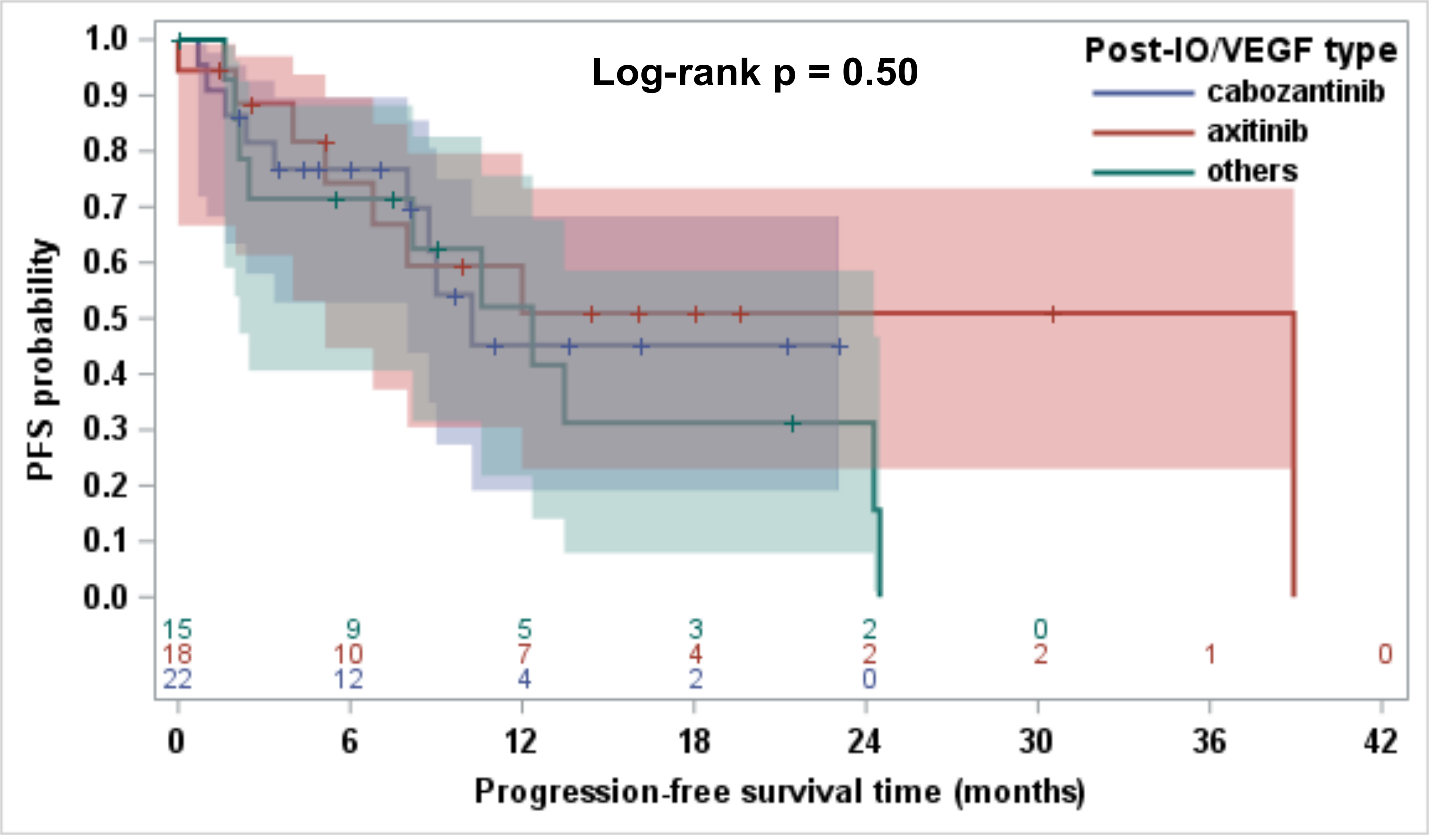


**Figure S4**. Kaplan Meier curve estimating PFS for 55 patients from the next line of systemic therapy for kidney cancer after discontinuation of IO-VEGF by subsequet therapy: Cabozatinib vs. Axitinib vs. Other agents with no statistical difference among the three groups (Log-rank p = 0.50). PFS (Progression Free Survival), IO-VEGF (Immune-Oncology and Vascular Endothelial Growth Factor targeted therapy).

| **Table S1. Best Objective response rate on next line of therapy post IO-VEGF by prior IO-VEGF combination** | | | |
| --- | --- | --- | --- |
| Type of response | IO-TKI (n=21) | IO-Bev (n=31) | Fisher’s exact p=0.76 |
| CR/PR | 5 (24%) | 9 (29%) |  |
| SD/PD | 16 (76%) | 22 (71%) |  |
| IO-VEGF (Immune-Oncology and Vascular Endothelial Growth Factor targeted therapy), IO-TKI (IO-Tyrosine Kinase Inhibitor), IO-Bev (IO-Bevacizumab), CR (Complete response), PR (Partial response), SD (Stable disease), PD (Progressive disease) | | | |

| **Table S2. Best Objective response rate on next line of therapy post IO-VEGF by type of next line received** | | | | |
| --- | --- | --- | --- | --- |
| Type of response | Cabozatinib (n=22) | Axitinib (n=16) | Other agents (n=14) | Fisher’s exact p=0.79 |
| CR/PR | 7 (32%) | 9 (22%) | 3 (21%) |  |
| SD/PD | 15 (68%) | 22 (71%) | 11 (79%) |  |
| IO-VEGF (Immune-Oncology and Vascular Endothelial Growth Factor targeted therapy), CR (Complete response), PR (Partial response), SD (Stable disease), PD (Progressive disease) | | | | |
